# Alfalfa Intervention Alters Rumen Microbial Community Development in Hu Lambs During Early Life

**DOI:** 10.3389/fmicb.2018.00574

**Published:** 2018-03-27

**Authors:** Bin Yang, Jiaqing Le, Peng Wu, Jianxin Liu, Le L. Guan, Jiakun Wang

**Affiliations:** ^1^Institute of Dairy Science, College of Animal Sciences, Zhejiang University, Hangzhou, China; ^2^Department of Agricultural, Food & Nutritional Science, University of Alberta, Edmonton, AB, Canada; ^3^ZJU-UoA Joint Laboratory for Livestock Functional Genomics and Microbiology, Zhejiang University, Hangzhou, China

**Keywords:** Hu lamb, rumen microbiota, starter, alfalfa intervention, amplicon sequencing

## Abstract

The pre-weaning period is crucial for rumen developmental plasticity, which can have a long-term impact on animal performance. Understanding the rumen microbiota during early life is important to elucidate its potential role in rumen development. In this study, the rumen microbiota of 10-day-old Hu lambs fed either milk replacer (B-10), milk replacer and starter (STA) or milk replacer and starter supplemented with alfalfa (S-ALF) in the pre- (d17, 24, and 38) and post-weaning periods (d45 and 66) were assessed to characterize rumen microbial colonization during early life and its response to fiber intervention. In the rumens of B-10 lambs, 498 operational taxonomic units belonging to 33 predominant genera were observed, and the top six predicted functions included “Membrane transport,” “carbohydrate metabolism,” “amino acid metabolism,” “replication and repair,” “translation,” and “energy metabolism.” *Prevotella*, *Succinivibrio*, *Bifidobacterium*, and *Butyrivibrio* abundances were increased at d38 for both STA and S-ALF groups compared to the B-10 group, whereas fibrolytic bacteria of the taxa *Lachnospiraceae* and *Treponema* were only increased in the S-ALF group at d38. A number of saccharolytic bacteria (*Bacteroidaceae*), organic acid-producing bacteria (*Coprococcus* and *Actinomyces*), proteolytic and amino acid fermenters (*Fusobacterium*) and fibrolytic bacteria (unclassified *Ruminococcaceae*) were significantly decreased in the STA lambs but not in the S-ALF lambs at d38. After weaning and exposed to alfalfa, the rumen microbial composition in the STA group started to appear similar to that of the S-ALF lambs. The relative abundance of unclassified *Clostridiales* was higher in S-ALF lambs than STA lambs after weaning. Spearman’s correlation analysis showed positive relationships between unclassified *Lachnospiraceae*, unclassified *Clostridiales*, *Treponema*, unclassified *Bacteroidales*, *Coprococcus* and crude protein intake, neutral detergent fiber intake, and plasma β-hydroxybutyrate. The unclassified *Lachnospiraceae* and *Treponema* were also positively correlated with average daily gain. Our results revealed that alfalfa stimulated changes in rumen microbiota during the pre- and post-weaning periods and was consistent with rumen development for better feed intake and animal performance before and after weaning. The findings of this study provide clues for strategies to improve rumen function through manipulation of the rumen microbiota during early life.

## Introduction

Early life, especially the pre-weaning period, is a critical period for the developmental plasticity of mammals and can have a long-term impact on various biological functions (Fisher et al., 2012; Bartol et al., 2013; Soberon and Van Amburgh, 2013). Within the first few weeks after birth, young ruminants face a weaning transition and dietary changes from milk or milk replacer to a solid diet. With the change in diet, the gastrointestinal tissues must transition from metabolizing glucose from milk to short-chain fatty acids from a solid diet, especially in the rumen (Baldwin et al., 2004). The weaning transition results in tremendous gastrointestinal and metabolic ramifications for the calf/lamb growth rate (Budzynska and Weary, 2008; de Passillé et al., 2011).

A starter diet containing highly fermentable carbohydrate has been widely used to feed young pre-weaned ruminants due to its ability to promote rumen development by enhancing rumen fermentation, primarily volatile fatty acid (VFA) production (Baldwin et al., 2004; Heinrichs, 2005; Suárez et al., 2006). Past research has revealed that the physical characteristics of feed, such as the particle sizes of roughages, can contribute to ruminal muscular development and size expansion (Tamate et al., 1962). A recent study observed that alfalfa supplementation to starter diets during the pre-weaning period increased rumen papillae length and rumen weight, decreased the incidence of feed plaques, and consequently led to increased feed intake, average daily gain (ADG), and carcass weight during the pre- and post-weaning periods (Yang et al., 2015). In addition, microbial colonization can also affect rumen development and function during early life. Using next generation DNA sequencing techniques, Li et al. (2012) and Jami et al. (2013) observed that prior to weaning, the ruminal microbiota has a similar functional capacity as that of a mature ruminant. Rey et al. (2012) confirmed these findings by measuring enzyme activities in the rumens of dairy calves from birth through weaning. Jami et al. (2013) deduced that the appearance of microbial populations during early life is not dependent on nutrient digestion with limited functional capacity but that it may play a role in long-term imprinting of the microbial community. Such speculation was supported by observations of reduced methane emissions in adult lambs after altering the methanogen community during the pre-weaning phase (Abecia et al., 2014). Based on these findings, we hypothesized that the positive effects of alfalfa supplementation to starter diets on both pre- and post-weaned lambs could be due to the impact on rumen microbial colonization. Therefore, in this study, we assessed the rumen microbiota of Hu lambs fed either a starter or a starter supplemented with alfalfa from the pre- to post-weaning period with the aim of characterizing the effects of alfalfa intervention on rumen microbial colonization during early life.

## Materials and Methods

### Animal Study and Sample Collection

All the experimental protocols performed in this study were approved by the Animal Care Committee of Zhejiang University (Hangzhou, China), and the experimental procedures used in this study were in accordance with the recommendations of the University’s guidelines for animal research.

In a previously published animal study (Yang et al., 2015), rumen samples were collected from Hu lambs without separating the solid and liquid fractions. Briefly, 66 healthy male Hu lambs at d5 [body weight (BW) = 3.69 ± 0.67 kg (mean ± SD)] were purchased and housed at the University research facility and fed milk replacer for 5 adaptation days before the feeding trial. At d10, six lambs were sacrificed as the baseline group (B-10), and the other 60 animals were randomly assigned to one of two diets (STA or S-ALF) and sacrificed at the age of d17, 24, 38, 45, or 66. Six lambs were assigned to each group for each sampling age. From d10 to d38 (pre-weaning), the lambs in the STA group were fed milk replacer and *ad libitum* starter pellets, whereas the lambs of the S-ALF group were provided the same starter diet with supplemental *ad libitum* chopped alfalfa. After weaning (from d38 to d66), all the lambs were fed 300 g/d of a concentrate mixture (Supplementary Table S1) and *ad libitum* alfalfa. BW was measured on two consecutive days before morning feeding in the beginning of the experiment (d10, initial BW), before sacrifice (end BW) and every week to calculate the ADG. Daily feed and ort samples were collected for chemical analysis to determine the intake of crude protein (CP) and neutral detergent fiber (NDF). Lambs were sacrificed before morning feeding, with plasma obtained before sacrifice and rumen tissues collected after sacrifice to measure the concentration of β-hydroxybutyrate (BHBA) and ruminal papillae length and width. Detailed information was published in Yang et al. (2015), with the exception that the data collected on the week of animal sacrifice for bacterial analysis were used in the present study.

The rumen content samples were collected immediately after sacrifice and stored at -20°C until further analysis. Only liquids were obtained from the rumen of the B-10 lambs, whereas mixed liquid and solid contents were obtained from the rumen of the STA and S-ALF lambs. After filtering out the lambs without solid feed intake (only milk replacer in the rumen) or lambs for which no rumen content was present after sacrifice, with the exception of the B-10 lambs (Yang et al., 2015), 55 valid rumen samples were acquired (6 samples from the B-10 group; 25 samples from the STA group, including 4, 3, 6, 6, and 6 samples at d17, 24, 38, 45, and 66, respectively; and 24 samples from the S-ALF group, including 4, 4, 5, 6, and 5 samples at d17, 24, 38, 45, and 66, respectively).

### Total DNA Extraction, Illumina Sequencing, and Data Processing

Total DNA from the rumen content samples was extracted using the cetyltrimethylammonium bromide method (Brookman and Nicholson, 2005) with a bead-beater (Biospec Products; Bartlesville, OK, United States) as described by Gagen et al. (2010). The amplicon library of the V4 hypervariable region of the 16S rRNA gene was prepared from each of the DNA samples using the primer set 515F/806R and Phusion^®^ High-Fidelity PCR Master Mix (New England Biolabs, Ipswich, MA, United States) as described by Caporaso et al. (2011). Each forward and reverse primer had a 6-bp error-correcting barcode at the 5′ terminus that was unique to each DNA sample. The amplicon libraries for all samples were pooled at an equimolar ratio and sequenced on an Illumina HiSeq platform by Novogene Bioinformatics Technology Co., Ltd. (Tianjin, China) to generate 2 × 250 bp paired-end reads.

The paired-end reads were joined to form single sequences using FLASH (Magoč and Salzberg, 2011) based on overlapping regions. The sequences were demultiplexed and assigned to each sample according to the individual unique barcode using Quantitative Insight into Microbial Ecology (QIIME, Caporaso et al., 2010). Sequences with a quality score of <20 and a length of >300 bp or <200 bp were discarded. Possible chimeric sequences were identified and removed using the usearch61 algorithm in USEARCH 6.1 (Edgar, 2010) with the Gold database^1^. Operational taxonomic units (OTUs) were clustered at a 97% identity threshold, and taxa were assigned using the core set in the Greengenes 13.8 database (DeSantis et al., 2006) using the UCLUST algorithm (Edgar, 2010). The alpha diversity of the ruminal bacteria was estimated using the number of OTUs, Chao1, Shannon indices, and Good’s coverage implemented in QIIME (Caporaso et al., 2010). Analysis of similarity (ANOSIM) was used to test whether a significant difference existed between two groups of samples. An *R*-value > 0.75 with a *P*-value < 0.05 denotes groups that are completely different from one another; 0.5 < *R*-value < 0.75 with a *P*-value < 0.05 denotes groups that are different from one another; 0.3 < *R*-value < 0.5 with *P*-value < 0.05 denotes groups that tend to be different from one another; and an *R*-value < 0.3 denotes groups that are not different from one another.

### Predicted Microbial Functions Using PICRUSt

PICRUSt (Phylogenetic Investigation of Communities by Reconstruction of Unobserved States, Langille et al., 2013) was used to predict the functional capabilities of microbial communities based on the 16S rRNA gene data against a Greengenes reference taxonomy (Greengenes 13.8). Briefly, after the abundance of each OTU was normalized by marker gene copy number, the molecular functions were predicted by the KEGG pathways.

### Statistical Analysis

All the data were subjected to a normal distribution test using SPSS 20.0 (SPSS, Inc., Chicago, IL, United States). Normally distributed data included alpha diversity indices (number of OTUs, Chao1, and Shannon index) and the phenotypic data (CP and NDF intake, initial and end BW, ADG, plasma BHBA concentration, and rumen papillae length and width). For the alpha diversity indices, an orthogonal polynomial regression analysis was performed to analyze the linear and quadratic effects of age from d10 to d38 and from d10 to d66 with Tukey’s multiple comparison test of the mean values performed among ages; one-way ANOVA was performed to analyze differences between the STA and S-ALF groups. For the phenotypic data one-way ANOVA was performed to analyze the age effect from d17 to d66 and the difference between the STA and S-ALF groups at different ages.

The non-normally distributed data included the relative abundance of rumen bacteria and the predicted KEGG pathway relative abundance. For the relative abundances of rumen bacteria, all the taxa analyzed in the present study were identified in at least 4 lambs at each assayed age for each feeding group (4 out of 6 lambs; except for d24 of STA group, which were identified in 3 lambs), with an average relative abundance of ≥0.5% in at least one age group (data were shown in Supplementary Table S2). This result meant that only the “core” bacteria that were observed in at least 60% of samples were considered in the relative abundance analysis. Taxa identified in each sample within each group but with an abundance <0.5% were not considered “core” bacteria. To show the changes in bacterial relative abundances, bacterial data were presented as log_2_ (fold change of d17-38 to B-10) during the pre-weaning period, and log_2_ (fold change of d45-66 to d38) during the post-weaning period, with taxa subjected to LEfSe analysis (Segata et al., 2011). A significant change was observed with a LDA (Linear Discriminant Analysis) score > 2.0 calculated by LEfSe. For the KEGG pathway relative abundance generated by PICRUSt, a Kruskal–Wallis signed rank test was performed in SPSS 20.0. A significant change was observed with *P* ≤ 0.05.

Spearman’s rank correlations between the ruminal bacteria (relative abundance) and CP intake, NDF intake, ADG, concentration of plasma BHBA, rumen papillae length and width were analyzed using SPSS 20.0, and only the significant correlations (*P* ≤ 0.05) of the changes in bacteria were plotted by R software (version 3.3.0) and the package “corrplot”.^2^

### Accession Number(s)

The paired-end sequence data were deposited and are available in the European Nucleotide Archive with the accession numbers ERS1929787-ERS1929792, ERS1485434-1485443, ERS1485445-1485470, ERS1485472-1485482, ERS1509216, and ERS1509217.

## Results

### Changes in Rumen Bacterial Diversity During Early Life of Hu Lambs in the STA and S-ALF Groups

A total of 1,458,338 qualified sequences were obtained from 55 rumen samples with an average of 32,734 ± 10,713 sequences per sample, and 1,682 operational taxonomic units (OTUs, 579 ± 85 OTUs per sample) were detected based on 97% similarity. With a subsample of 23,600 sequences (the minimum number detected) for each sample, the Good’s coverage (>0.98) revealed that our data provided sufficient sequencing depth to accurately describe the rumen bacterial composition of the Hu lambs used in this study.

In the rumens of the B-10 Hu lambs, 498 OTUs were identified with a Shannon index of 5.29 (**Table 1**). When considering the whole experimental period from d10 to d66, the number of OTUs and the Shannon index increased linearly for lambs in both the STA and S-ALF groups (*P* < 0.05, **Table 1**), and no significant difference (*P* > 0.05) was observed between lambs in the two groups for each age. However, the alpha diversity patterns for the lambs in the two groups were different during the pre- and post-weaning periods. The number of OTUs increased quadratically (*P* < 0.05) from d10 to d38 for lambs in the STA group, and the highest number of OTUs appeared on d17. After weaning, the number of OTUs and the Shannon index increased significantly (*P* < 0.05) when compared with the values observed on d38 for lambs of the STA group, whereas these two indices were not significantly higher on d45 than d38 for lambs in the S-ALF group (**Table 1**). At d66, the number of OTUs and the Shannon index for lambs in the STA group increased to 637 ± 44 and 6.68 ± 0.29, respectively, and these two indices for S-ALF lambs increased to 675 ± 77 and 7.11 ± 0.69 (**Table 1**).

**Table 1:** Alpha diversity (mean ± SD) of rumen bacterial communities in Hu lambs with (S-ALF) or without (STA) alfalfa intervention at different ages.

	STA			S-ALF			STA vs. S-ALF^2^		
Age (d)	OTUs	Chao1	Shannon	OTUs	Chao1	Shannon	OTUs	Chao1	Shannon
10	498 ± 60^b^	1028 ± 98	5.29 ± 0.67^bc^	498 ± 60^b^	1028 ± 98	5.29 ± 0.67^b^	–	–	–
17	566 ± 57^ab^	1016 ± 110	5.33 ± 0.60^bc^	558 ± 72^ab^	1007 ± 75	5.50 ± 0.89^ab^	0.868	0.907	0.759
24	554 ± 45^ab^	1044 ± 81	5.33 ± 0.20^bc^	625 ± 127^ab^	1070 ± 66	5.94 ± 1.38^ab^	0.407	0.660	0.496
38	487 ± 36^b^	946 ± 98	4.86 ± 0.31^cd^	566 ± 100^ab^	1007 ± 108	5.61 ± 0.89^ab^	0.102	0.354	0.086
45	586 ± 73^a^	1038 ± 95	6.14 ± 0.92^ab^	626 ± 31^ab^	1062 ± 42	6.65 ± 0.54^ab^	0.244	0.574	0.263
66	637 ± 44^a^	1052 ± 67	6.68 ± 0.29^a^	675 ± 77^a^	1135 ± 118	7.11 ± 0.69^a^	0.331	0.177	0.190
Age effect^1^									
From d10 to 38								
Linear	0.641	0.260	0.201	0.144	0.994	0.469	–	–	–
Quadratic	0.014	0.380	0.314	0.182	0.628	0.551	–	–	–
From d10 to 66								
Linear	0.002	0.795	<0.001	0.002	0.063	0.001	–	–	–
Quadratic	0.118	0.316	0.004	0.805	0.242	0.293	–	–	–

*^*a*-*c*^Means within a column with different superscripts differ (*P*-value ≤ 0.05). ^*1*^Linearly and quadraticly effect of age during the pre-weaning period (from d10 to 38), and the whole period (from d10 to 66). ^*2*^*P*-value of the comparison between STA and S-ALF group.*

### Succession of Rumen Bacterial Communities During the Early Life of Hu Lambs in the STA and S-ALF Groups

Similar to the observed changing alpha diversity patterns, the analysis of similarity (ANOSIM) results showed that the rumen microbial communities were similar among the B-10, d17 and d24 lambs before weaning and between d45 and d66 lambs after weaning, and there was no significant difference between lambs of the two groups at each age (*R* < 0.3 with *P* > 0.05, **Table 2**). For the STA group, the rumen microbial communities in the d38 animals were different from those observed in the B-10 and STA d17 and d24 lambs (0.5 < *R* < 0.75 with *P* < 0.05), and the microbial communities were significantly shifted after weaning. Compared to the d38 STA lambs, the *R*-value of ANOSIM was 0.887 (*P* < 0.05) for d45 and 1.000 (*P* < 0.05) for d66 STA lambs (**Table 2**). For the S-ALF group, the microbial communities in d38 lambs tended to be different from those in the B-10 and STA d17 and d24 lambs (0.3 < *R* < 0.5 with *P* < 0.05), and this tendency was observed at d45 after weaning (**Table 2**). Compared to the d38 S-ALF lambs, the *R*-value of ANOSIM was 0.403 (*P* < 0.05) for d45 and 0.648 (*P* < 0.05) for d66 S-ALF lambs (**Table 2**).

**Table 2:** *R*- and *P*-values of pairwise comparison between the different ages in the groups with (S-ALF) or without (STA) alfalfa intervention performed using analysis of similarity (ANOSIM)^1^.

*R*-values	B-10	STA-17	STA-24	STA-38	STA-45	STA-66	S-ALF-17	S-ALF-24	S-ALF-38	S-ALF-45	S-ALF-66
B-10	0										
STA-17	0.298	0									
STA-24	0.173	-0.093	0								
STA-38	0.591*	0.385*	0.531*	0							
STA-45	0.550*	0.504*	0.543*	0.887*	0						
STA-66	0.624*	0.794*	0.815*	1.000*	0.150	0					
S-ALF-17	0.024	0.031	0.074	0.738*	0.468*	0.706	0				
S-ALF-24	0.302	0.125	-0.130	0.690*	0.361*	0.496	0.208	0			
S-ALF-38	0.440*	0.094	0.405	0.035	0.523*	0.688	0.462*	0.394*	0		
S-ALF-45	0.559*	0.464*	0.469*	0.722*	0.102	0.028	0.488*	0.282*	0.403*	0	
S-ALF-66	0.419*	0.587*	0.579*	0.976*	0.133	0.091	0.388*	0.106	0.648*	0.021	0

*^*1*^*R*-value > 0.75 with *P*-value < 0.05 denote groups completely different from one another; 0.5 < *R*-value < 0.75 with *P*-value < 0.05 denote groups different from one another; 0.3 < *R*-value < 0.5 with *P*-value < 0.05 denote groups tend to be different from one another; *R*-value < 0.3 denote groups not different from one another. ^∗^Represents the pairwise comparison with *P*-value < 0.05.*

### Divergence of the Rumen Bacterial Communities During the Pre-weaning Period of Hu Lambs in the STA and S-ALF Groups

#### Rumen Microbial Composition of Milk Replacer-Fed Hu Lambs

Seven predominant phyla were identified in the rumens of the B-10 lambs. Among them, *Bacteroidetes*, *Firmicutes* and *Proteobacteria* were the dominant phyla and accounted for 41.5, 35.5, and 17.8% of the total sequences, respectively, followed by *Fusobacteria* (1.7%), *Actinobacteria* (1.4%), *Verrucomicrobia* (1.1%) and *Chloroflexi* (0.6%) (Supplementary Table S3). Thirty-three predominant genera were identified in the rumens of the B-10 lambs, with *Bacteroides* (20.9%) being the most abundant; the relative abundances of unclassified *BS11*, *Prevotella*, and *Dialister* were between 5 and 10%; unclassified *Lachnospiraceae*, unclassified *Clostridiales*, *Eikenella*, *Sharpea*, *Porphyromonas*, unclassified *Pasteurellaceae*, unclassified *Ruminococcaceae*, *Bibersteinia*, *Fusobacterium*, *Mitsuokella*, *Coprococcus*, *Megasphaera*, *Oscillospira*, unclassified *Enterobacteriaceae*, *Butyrivibrio*, *Streptococcus*, *Akkermansia*, unclassified *Mogibacteriaceae*, and *Lactobacillus* were between 1 and 5%; and unclassified *Bacteroidales*, *Moraxella*, unclassified *S24-7*, *Ruminococcus*, *CF231*, unclassified *Coriobacteriaceae*, *Sutterella*, *SHD-231*, unclassified *Aeromonadaceae*, and unclassified *Veillonellaceae* were less than 1% (Supplementary Table S4).

#### Microbial Compositional Changes in the Rumen of STA-Fed Lambs

**FIGURE 1:**
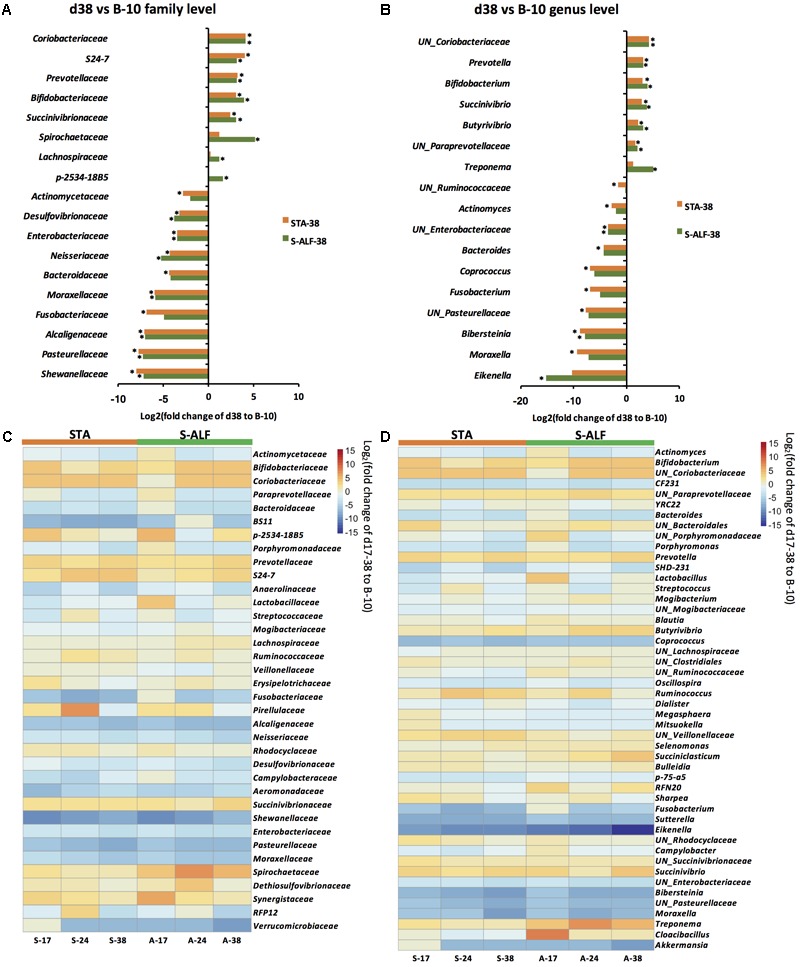
Succession of rumen bacterial communities during the pre-weaning period in the groups with (S-ALF) or without (STA) alfalfa intervention. **(A)** Family level changes between the 10-day baseline group (B-10) and the STA and S-ALF groups at end of the pre-weaning period (d38). **(B)** Genus level changes between B-10 and the d38 lambs. **(C)** Bacterial changes at the family level from the d10 to d38 lambs. **(D)** Bacterial changes at the genus level from d10 to d38. ^∗^ in **(A)** and **(B)** represents significant difference compared to the B-10 group. UN denotes an unclassified genus.

**Figure 1** shows the taxa that significantly (*P* ≤ 0.05) changed in abundance during the pre-weaning period. Compared to the B-10 group, 15 core families and 15 core genera were altered in the STA group on d38 (**Figures 1A,B**). The families *Coriobacteriaceae*, *S24-7*, *Prevotellaceae*, *Bifidobacteraceae,* and *Succinivibrionaceae* and the genera *Prevotella*, *Bifidobacterium*, *Succinivibrio*, *Butyrivibrio*, unclassified genera within *Paraprevotellaceae* and *Coriobacteriaceae* increased significantly. In contrast, the families *Shewanellaceae*, *Pasteurellaceae*, *Alcaligenaceae*, *Fusobacteriaceae*, *Moraxellaceae*, *Bacteroidaceae*, *Neisseriaceae*, *Enterobacteriaceae*, *Desulfovibrionaceae*, and *Actinomycetaceae* and the genera *Moraxella*, *Bibersteinia*, *Fusobacterium*, *Coprococcus*, *Bacteroides*, *Actinomyces*, unclassified genera within *Pasteurellaceae*, *Enterobacteriaceae*, and *Ruminococcaceae* decreased significantly.

Although fluctuations in the bacterial relative abundances between the B-10 group and the STA lambs on d38, the 15 families and 15 genera that exhibited significant changes in the d38 animals compared to the B-10 group were observed to continuously increase or decrease from B-10 to d38 in the STA group (**Figures 1C,D**).

#### Microbial Compositional Changes in the Rumen of the S-ALF-Fed Lambs

Compared to the B-10 lambs, 15 core families and 10 core genera were altered in the S-ALF group on d38 (**Figures 1A,B**). In addition to the changes in taxa observed in the d38 STA lambs, the families *Spirochaetaceae*, *Lachnospiraceae*, and *p-2534-18B5* and the genus *Treponema* were additionally increased in the d38 S-ALF lambs. In contrast, the families *Fusobacteriaceae*, *Bacteroidaceae*, and *Actinomycetaceae* and the genera *Moraxella*, *Fusobacterium*, *Coprococcus*, *Bacteroides*, *Actinomyces*, unclassified genera within *Pasteurellaceae* and *Ruminococcaceae* were not significantly decreased at d38 in the S-ALF lambs. The relative abundance of *Eikenella* was significantly decreased at d38 in the S-ALF lambs.

These significantly changes in families and genera on in the S-ALF group on d38 compared to the B-10 group were continuously increased or decreased from B-10 to d38 (**Figures 1C,D**), except for families *Lachnospiraceae* and *p-2534-18B5*, with the family *Lachnospiraceae* increasing since d24 and the family *p-2534-18B5* was decreased at d24 (**Figure 1D**).

Comparing the relative abundances of taxa between the two groups of lambs, unclassified *Lachnospiraceae*, unclassified *Ruminococcaceae*, and *Oscillospira* were significantly higher in the rumen of the d38 S-ALF lambs than that in d38 STA lambs (Supplementary Figure S1).

### Effect of Pre-weaned Dietary Intervention on Rumen Microbial Adaptation to Weaning and Diet Transition

**FIGURE 2:**
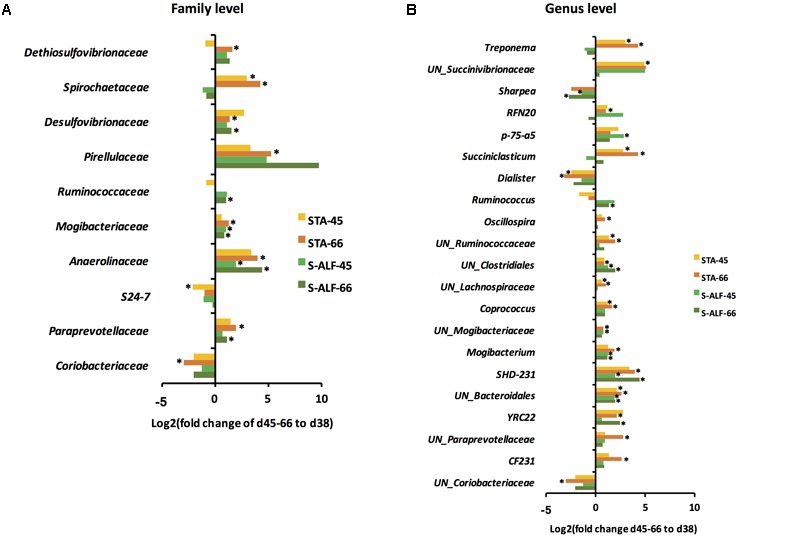
Changes in the rumen bacterial communities during the post-weaning period in lambs with (S-ALF) or without (STA) alfalfa intervention. **(A)** Family level changes between post-weaning (d45 and 66) and d38 lambs. **(B)** Genus level changes between post-weaning (d45 and 66) and d38 lambs. ^∗^Represents significant difference when compared with d38 age group. UN denotes an unclassified genus.

Although the rumen bacterial communities in the d45 and d66 lambs did not differ within or between the STA or S-ALF groups (**Table 2**), the succession of the rumen bacterial communities from d38 to the post-weaning period was different. **Figure 2** shows the significant changes in the families and genera of the d45 and d66 vs. d38 lambs for the STA and S-ALF groups. Weaning and diet transition significantly increased the relative abundances of *Paraprevotellaceae*, *Anaerolinaceae*, *Mogibacteriaceae* and *Desulfovibrionaceae* in both the STA and S-ALF groups (**Figure 2A**). Among these taxa, the significant increases in *Anaerolinaceae* and *Mogibacteriaceae* were observed in the S-ALF group since d45, while the significant increases of the 4 families in the STA group were observed at d66 (**Figure 2A**). Additionally, *Spirochaetaceae*, which was increased in the S-ALF lambs before weaning (**Figure 1A**), was increased in the STA lambs since d45 (**Figure 2A**). The *Coriobacteriaceae* was increased in both the STA and S-ALF groups before weaning (**Figure 1A**), but decreased after weaning, and this decrease was significant in the STA group on d66 (**Figure 2A**). The *Ruminococcaceae* was only significantly increased in the S-ALF group on d66 (**Figure 2A**). The *Dethiosulfovibrionaceae* and *Pirellulaceae* were only significantly increased in the STA groups on d66 (**Figure 2A**).

Weaning and diet transition significantly increased the *YRC22*, *SHD-231*, *Mogibacterium*, and unclassified genera within the taxa *Mogibacteriaceae*, *Bacteroidales* and *Clostridiales* in both the STA and S-ALF groups (**Figure 2B**). The relative abundances of unclassified *Lachnospiraceae*, unclassified *Ruminococcaceae*, and *Treponema*, which were increased in the S-ALF lambs before weaning (**Figure 1B**), were increased in the STA lambs since d45 (**Figure 2B**). The relative abundance of *Coprococcus*, which decreased with and without significance in the STA and S-ALF lambs at d38 (**Figure 1B**), increased significantly in the STA lambs since d45 (**Figure 2B**). The significantly increased *Ruminococcus* was only observed in S-ALF group on d66 (**Figure 2B**).

Comparing the relative abundances of taxa between the two groups of lambs, the unclassified *Mogibacteriaceae* was higher in the S-ALF lambs at d45; and the *SHD-231*, *Bibersteinia*, *Actinomyces*, and unclassified *Clostridiales* were higher, while the *Prevotella*, unclassified *Paraprevotellaceae*, and *Campylobacter* were lower in the S-ALF lambs at d66 (Supplementary Figure S1).

### Divergence of Predicted Rumen Microbial Functions in the STA and S-ALF Groups

**FIGURE 3:**
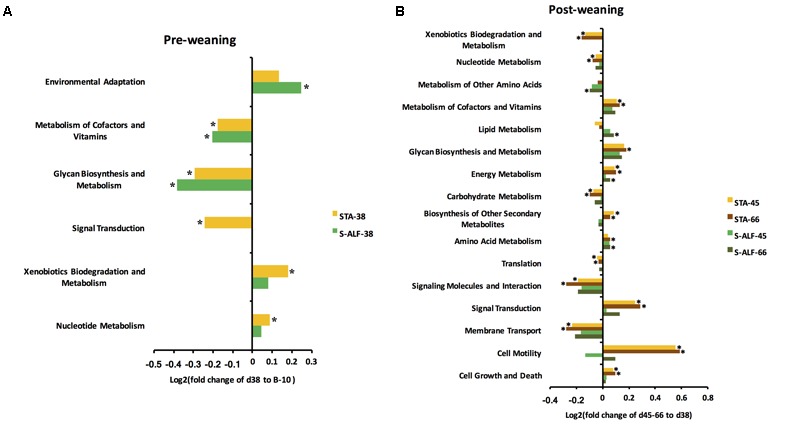
Changes in predicted metagenomic functions of rumen bacteria in lambs with (S-ALF) or without (STA) alfalfa intervention during the pre- **(A)** and post-weaning period **(B)**. ^∗^Represents a significant difference (*P* ≤ 0.05) when compared to B-10 during the pre-weaning period and when compared to the d38 lambs during the post-weaning period.

“Membrane transport” (11.1%), “carbohydrate metabolism” (10.5%), “amino acid metabolism” (10.0%), “replication and repair” (9.4%), “translation” (6.2%), and “energy metabolism” (5.9%) were identified as the top six predicted functions for the rumen microbiota in the B-10 lambs, which were also the top predicted functions for rumen microbiota in the STA and S-ALF lambs across all ages. In the STA lambs, the functions “nucleotide metabolism” and “xenobiotic biodegradation and metabolism” increased (*P* < 0.05), whereas “signal transduction,” “glycan biosynthesis and metabolism,” and “metabolism of cofactors and vitamins” decreased (*P* < 0.05) in d38 lambs compared to the B-10 group (**Figure 3A**). For the STA lambs, after weaning, “cell growth and death,” “cell motility,” “signal transduction,” “amino acid metabolism,” “biosynthesis of other secondary metabolites,” “energy metabolism,” “glycan biosynthesis and metabolism,” and “metabolism of cofactors and vitamins” increased (*P* < 0.05), whereas “membrane transport,” “signaling molecules and interactions,” “translation,” “carbohydrate metabolism,” “nucleotide metabolism,” and “xenobiotic biodegradation and metabolism” decreased (*P* < 0.05) compared to the d38 animals (**Figure 3B**).

For the S-ALF group, “environmental adaptation” increased while “glycan biosynthesis and metabolism,” and “metabolism of cofactors and vitamins” decreased at d38 compared to the B-10 group (*P* < 0.05, **Figure 3A**). After weaning, the changes in the S-ALF were less pronounced when compared to the STA lambs. Only “amino acid metabolism,” “energy metabolism,” and “lipid metabolism” increased (*P* < 0.05), and the “metabolism of other amino acids” decreased (*P* < 0.05) in d66 lambs compared to d38 lambs (**Figure 3B**).

### Relationship Between Bacterial Community and Phenotypic Variables

**FIGURE 4:**
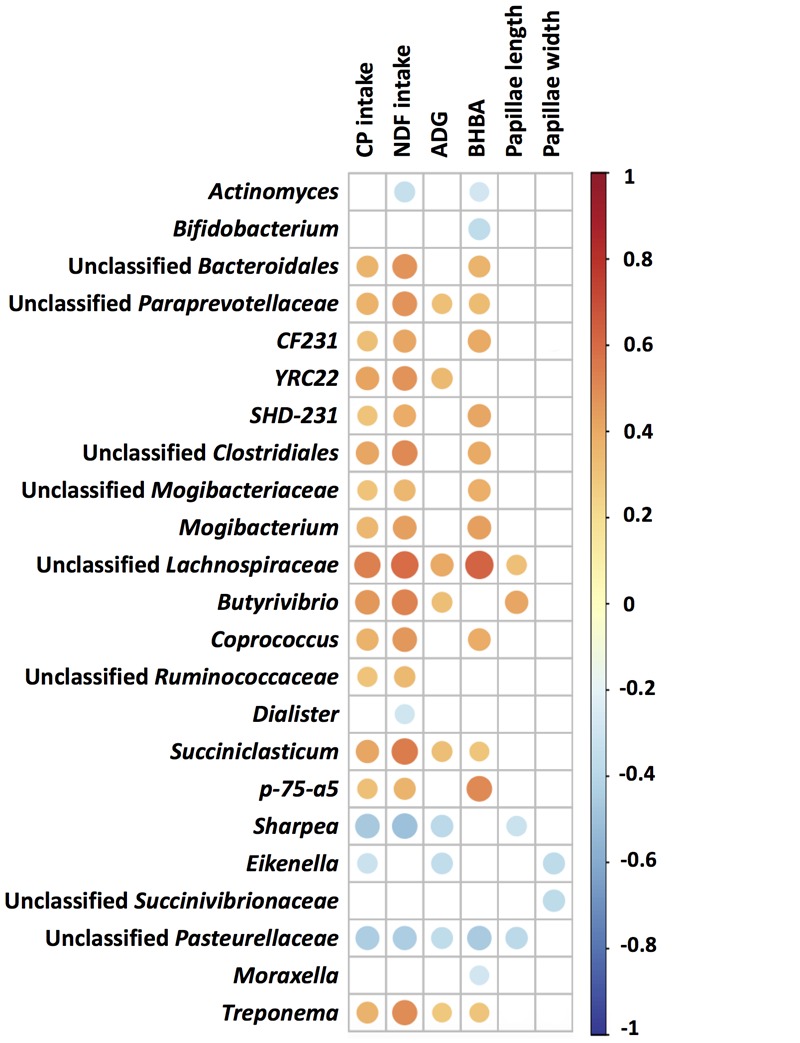
Spearman’s correlation between the rumen bacterial communities (genus level) and phenotypic variables. The color and dot size represent the correlation coefficient. Red represents a positive correlation, and blue represents a negative correlation; a larger dot size represents a stronger correlation, and a smaller dot size represents a weaker correlation. CP, crude protein; NDF, neutral detergent fiber; ADG, average daily gain; BHBA, β-hydroxybutyrate.

The CP and NDF intake, initial and end BW, ADG, plasma BHBA concentration, rumen papillae length and width were obtained from our previous study (Yang et al., 2015) and re-analyzed with data of 55 lambs for bacteria analysis only in the week of sacrifice (**Table 3**). Correlation analysis showed that the relative abundances of *CF231*, *YRC22*, *SHD-231*, *Mogibacterium*, *Butyrivibrio*, *Coprococcus*, *Succiniclusticum*, *p-75-a5*, *Treponema*, and unclassified genera within the *Bacteroidales*, *Paraprevotellaceae*, *Clostridiales*, *Mogibacteriaceae*, *Lachnospiraceae*, and *Ruminococcaceae* taxa were positively correlated with CP and NDF intake, while the relative abundances of *Sharpea* and unclassified *Pasteurellaceae* were negatively correlated with CP and NDF intake; *Eikenella* was negatively correlated with CP intake, and *Actinomyces* and *Dialister* were negatively correlated with NDF intake (*P* < 0.05, **Figure 4**). Significant correlations were also observed between these bacteria and rumen developmental parameters of plasma BHBA and ruminal papillae length or width, except for *YRC22* and unclassified *Ruminococcaceae*. In addition, *Bifidobacterium* and *Moraxella* were negatively correlated with plasma BHBA, and unclassified *Succinivibrionaceae* was negatively correlated with ruminal papillae width (*P* < 0.05, **Figure 4**). The relative abundances of the genera *YRC22*, *Butyrivibrio*, *Succiniclasticum*, *Treponema*, and unclassified genera within the families *Paraprevotellaceae* and *Lachnospiraceae* showed positive correlations with ADG, while the genera *Sharpea*, *Eikenella*, and unclassified genera within the family *Pasteurellaceae* showed negative correlations with ADG (*P* < 0.05, **Figure 4**).

**Table 3:** Phenotypic variables in Hu lambs of the groups with (S-ALF) or without (STA) alfalfa intervention at different ages.

		STA (d)							S-ALF (d)						
Items^1^	B-10	17	24	38	45	66	SEM^2^	*P*-value^2^	17	24	38	45	66	SEM^3^	*P*-value^3^
CP intake, g	19.57	25.30	20.96	81.80	54.29	126.85	8.630	<0.001	24.82	24.34	107.26^∗^	102.25^∗^	154.59^∗^	10.504	<0.001
NDF intake, g	3.85	6.33	6.37	54.03	85.33	197.08	15.242	<0.001	6.35	11.49	80.49^∗^	162.77^∗^	244.49^∗^	19.211	<0.001
Initial BW, kg	3.10	3.64	3.68	3.62	3.99	3.90	0.151	0.913	3.65	3.08	3.88	3.79	4.16	0.134	0.140
End BW, kg	3.10	3.80	3.95	6.18	7.05	12.18	0.679	<0.001	3.80	3.41	7.38	8.76	15.21^∗^	0.885	<0.001
ADG, kg	–	0.054	0.005	0.182	0.034	0.280	0.026	<0.001	0.050	0.032	0.251^∗^	0.183^∗^	0.268	0.022	<0.001
Rumen papillae															
Length, mm	0.383	0.508	0.484	1.474	1.028	1.329	0.086	<0.001	0.431	0.587^∗^	1.681	1.311^∗^	1.626^∗^	0.108	<0.001
Width, mm	0.171	0.209	0.226	0.268	0.207	0.234	0.011	0.311	0.208	0.233	0.323^∗^	0.209	0.251	0.013	0.006
BHBA, μmol/L	52.8	344.3	236.0	241.2	400.4	471.4	30.036	0.032	142.8	133.7	339.7	789.8^∗^	560.0	65.031	<0.001

*^*1*^CP, crude protein; NDF, neutral detergent fiber; Initial BW, body weight at d10; End BW, body weight before sacrifice; ADG, average daily gain; BHBA, β-hydroxybutyrate. ^*2*^SEM and *P*-value of the STA group from d17 to 66. ^*3*^SEM and *P*-value of the S-ALF group from d17 to 66. ^∗^Indicates that the item was significantly different in the S-ALF group (*P* ≤ 0.05) when compared to the same age of the STA group.*

## Discussion

The structure of the rumen microbiome during early life has recently attracted attention because of its potential relationship with rumen development (Li et al., 2012; Jami et al., 2013) and its long-term impact on an animal’s performance (Cahenzli et al., 2013; Abecia et al., 2014). Although the appearance of the microbial populations precedes rumen development, it has been suggested that the development of the rumen and its microbiota begins with the intake of solid feed (Rey et al., 2014). In the rumens of the B-10 lambs, the 498 observed OTUs belonged to 33 predominant genera and the top six predicted functions included “Membrane transport,” “carbohydrate metabolism,” “amino acid metabolism,” “replication and repair,” “translation,” and “energy metabolism.” These findings confirmed that the appearance of the metabolically related microbial populations precedes rumen development and is not dependent on solid feed intake as was previously suggested (Jami et al., 2013). The divergence of the rumen bacterial communities during the pre-weaning period of Hu lambs in the STA and S-ALF groups (**Figure 1** and **Table 2**), together with the significant correlations between bacteria and CP and NDF intake (**Figure 4**), further confirmed that changes in the rumen microbiota could in response to the intake of solid feed. Li et al. (2012) also showed that solid feed intake distinctly altered the rumen microbial composition.

Only 10–15 genera exhibited significant changes on d38 after solid feed intake (**Figure 1**). How can these microbes play such an important function in a redundant ruminal microbial environment? It was previously noted that significant microbial compositional changes may not lead to a functional shift because many microbes share the same metabolic pathways. Li et al. (2012) observed that all of the functional classes between two age groups (d14 and d42 of calves) were similar, suggesting that although their phylogenetic composition greatly fluctuated, the rumen microbial communities of pre-ruminant calves maintained a stable function and metabolic potentials. In our current study, the abundances of the genera *Prevotella* and *Butyrivibrio* increased the most, with increases in *Succinivibrio* and *Bifidobacterium* also observed at d38 when compared to the B-10 lambs. The members of the *Prevotella* genus are highly amylolytic and proteolytic (Matsui et al., 2000; Xu and Gordon, 2003). The genus *Butyrivibrio* represents the primary butyrate producers in the rumen and are considered effective hemicellulose degraders (Diez-Gonzalez et al., 1999; Paillard et al., 2007), and several species in *Butyrivibrio* are also responsible for their high proteolytic activity (Cotta and Hespell, 1986; Attwood and Reilly, 1995; Sales et al., 2000). *Succinivibrio* and *Bifidobacterium* are saccharolytic bacteria and can produce acetate and lactate (Bryant, 2015; Biavati and Mattarelli, 2015). The higher abundances of these genera suggests the potential increase in carbohydrate and protein metabolism in the rumen with increased starter intake. In addition, the increased concentration of ruminal butyrate before weaning was consistent with the relative abundance of *Butyrivibrio* (Yang et al., 2015). However, the predicted functions of the carbohydrate and amino acid metabolism by PICRUSt were not significantly changed during the pre-weaning period. Although PICRUSt has been demonstrated to be a useful tool to predict the function of microbiota from various environments based on 16S rRNA gene sequences (Langille et al., 2013), this tool was developed based on Greengenes (DeSantis et al., 2006) and IMG (the integrated microbial genomes database and comparative analysis system, Markowitz et al., 2011). Due to the nature of the rumen microbiota, many of the functions of unclassified bacteria may be underestimated. To further understand the impact of increased bacterial taxa on rumen function, functional analysis of the rumen microbiome using metagenomic and/or metabolomic analyses should be integrated.

The fibrolytic bacteria of the taxa *Lachnospiraceae* (Biddle et al., 2013) and *Treponema* (Ziołecki, 1979) were only increased in the S-ALF group at d38. The relative abundances of some saccharolytic bacteria (such as family *Bacteroidaceae*) (Song et al., 2015), short-chain fatty acid producers (such as *Coprococcus* and *Actinomyces*) (Buchanan and Pine, 1962; Tsai et al., 1976), proteolytic fermenters (such as *Fusobacterium*) (Takahashi, 2003), and fibrolytic bacteria of unclassified *Ruminococcaceae* (Brulc et al., 2009; Biddle et al., 2013; Nyonyo et al., 2014) were significantly decreased in the STA lambs but not in the S-ALF lambs before weaning (**Figures 1B,D**). These results occurred because of the bigger variation among individual S-ALF lambs than was observed in the STA group, likely as an effect of alfalfa intervention. After weaning and dietary transition, the abundances of *Treponema*, unclassified *Lachnospiraceae*, and unclassified *Ruminococcaceae*, which had higher relative abundances in the S-ALF lambs before weaning, began to increase in the STA group (**Figure 2B**). It was previously reported that *Treponema* were closely associated with pectin-rich treatments due to the ability of species of this genus to degrade pectin (Liu et al., 2015). Alfalfa contains a pectin content of 10.5–14.2% (Mertens, 2003), which is more than grass and corn stover (Waite and Gorrod, 1959; Mullen et al., 2010). Therefore, in the present study, the increase in *Treponema* was a response to alfalfa intake, suggesting that the intervention of alfalfa had an effect on the rumen microbial composition. After weaning, when both groups were exposed to alfalfa, the microbial composition in the STA group began to approach that of the S-ALF lambs. Furthermore, after weaning, the relative abundances of unclassified *Clostridiales* were higher in S-ALF lambs than in the STA lambs, and *Ruminococcus* was only increased in S-ALF lambs. The order *Clostridiales* includes many polysaccharolytic bacteria that contribute to the production of VFAs in the gut (Chinda et al., 2004). Some *Ruminococcus* strains are cellulolytic fiber-degrading bacteria (Ezaki, 2015) with cellulosome systems (Ben David et al., 2015). Members of this genus are all organic acid-producing bacteria relating to fiber digestion. These results suggested the positive effects of alfalfa supplementation on microbial changes and might improve rumen digestion. The microbial changes that occurred in response to weaning transition and dietary changes have been previously reported (Li et al., 2012; Jami et al., 2013; Oikonomou et al., 2013; Meale et al., 2016), but these studies used dairy calves. Studies by Oikonomou et al. (2013) and Meale et al. (2016) focused on fecal microbiota, and the other two studies only reported rumen microbial changes at the phylum level, and none of these studies compared post-weaning changes after dietary interventions. Therefore, our study is a more comprehensive assessment of rumen microbial colonization, taking into account the adaptation of the microbiota to weaning transition and dietary changes and how the dietary intervention can potentially manipulate this process.

Butyrate was shown to be an important regulator and stimulator of development of the rumen (Górka et al., 2011) and small intestine (Guilloteau et al., 2009) in calves. Members of the genus *Butyrivibrio* represents the primary butyrate producers in the rumen (Bryant, 1986) and a positive relationship between *Butyrivibrio* and papillae length in the rumen was observed in our correlation analysis (**Figure 4**). Plasma BHBA is a parameter that is associated with the physical development of the rumen (Quigley et al., 1991; Khan et al., 2011). The positive correlation between the unclassified *Lachnospiraceae* and unclassified *Clostridiales* and BHBA, the higher abundance of unclassified *Lachnospiraceae* in the S-ALF lambs before weaning, and the significant increase of unclassified *Clostridiales* in S-ALF lambs that occurred shortly after weaning suggested the positive effects of alfalfa supplementation on microbial changes, which may promote rumen physical development. Acetate is a VFA that provide energy for the host through its conversion to ketone bodies (Pennington, 1952). Unclassified *Lachnospiraceae* and unclassified *Clostridiales* are the major producers of acetate (Ezaki, 2015). The concentration of acetate in the rumen did not differ between the STA and S-ALF lambs at each assayed age (Yang et al., 2015), while a higher (*P* ≤ 0.05) BHBA concentration in the plasma was observed in the S-ALF lambs after weaning (**Table 3**). Therefore, higher amounts of acetate, which is quickly absorbed by the rumen, were not detected in the rumens of the S-ALF lambs compared with those of the STA lambs. Previous studies on the positive effect of fiber supplementation on the performance of young ruminants (Terré et al., 2013; Mirzaei et al., 2015) were primarily focused on the physical stimulation and/or chemical nutrition of the forage (Beiranvand et al., 2014; Terré et al., 2015), whereas our current study is the first to investigate the role of the rumen microbiota. Taken together, the results of previous studies and our current study suggest that the supplementation of fibers can influence rumen development through physical, chemical and microbial mechanisms.

The abundances of *Eikenella* and *Campylobacter* were lower in the rumens of the S-ALF lambs before and after weaning, respectively. Bacteria belonging to these genera have been associated with a variety of veterinary diseases (Behling et al., 1979; Humphrey and Beckett, 1987). The growth or persistence of *Campylobacter jejuni* in the rumen was supported by the data of Stanley et al. (1998a,b), especially in the rumens of young animals (Stanley and Jones, 2003). The observed decrease in *Eikenella* and *Campylobacter* indicated the potential impact of the alfalfa intervention on reducing the presence these genera, which could reduce the incidence of diseases and their shedding into the environment.

In summary, alfalfa supplementation with starter stimulates the proliferation of fibrolytic bacteria, including unclassified *Lachnospiraceae* and *Treponema*, and promoted the presence of some saccharolytic bacteria and short-chain fatty acid producers prior to weaning. Positive relationships were observed between unclassified *Lachnospiraceae*, *Treponema* and nutrient intake, ADG, and plasma BHBA, although the causal relationship between the host and microbiota is still unclear. While limitations of our study included that the rumen samples were collected by slaughter and comparisons of the ages and diet effects were not performed from the same lambs, the significantly changes in taxa between the B-10 group and the d38 STA and S-ALF lambs might allow the causal effect relationship of the host and microbiota to be determined. The microbial transplantation of d38 STA or S-ALF microbiotas into B-10 lambs or treating the rumen epithelial cell culture with ultrafiltered rumen fluid from lambs from these groups should provide more direct evidence on whether the identified microbial taxa changes could influence rumen development. Regardless, our findings suggest that after colostrum intake, a milk replacer and *ad libitum* starter pellets supplemented with alfalfa are recommended for the early weaning system to improve young ruminant health and performance.

## Author Contributions

All of the authors contributed intellectual input and assisted with this study and manuscript. JW and BY designed the study and collected content samples. BY and JQL extracted the rumen content DNA. BY and PW contributed to the data analysis; and BY, JXL, LG, and JW prepared the manuscript. All of the authors have read and approved the manuscript.

## Conflict of Interest Statement

The authors declare that the research was conducted in the absence of any commercial or financial relationships that could be construed as a potential conflict of interest.

## Footnotes

1 https://drive5.com/uchime/uchime_download.html

2 https://CRAN.R-project.org/package=corrplot
