# Supplementary material for: Alfalfa Intervention Alters Rumen Microbial Community Development in Hu Lambs During Early Life
Source: Front Microbiol. 2018 Mar 27;9:574. doi: 10.3389/fmicb.2018.00574 (PMC5881016; doi:10.3389/fmicb.2018.00574)
Supplement: Supplementary file 1 [file Table_1.DOCX]

**Table S1. Components of concentrate mixture fed to lambs during the post-weaning period.**

| Components | Proportion, % |
| --- | --- |
| Corn grain | 45 |
| Cottonseed cake | 20 |
| Soybean meal | 15 |
| Wheat bran | 15 |
| NaCl | 2 |
| NaHCO_3_ | 1 |
| CaHPO_4_ | 2 |
