# Supplementary material for: Alfalfa Intervention Alters Rumen Microbial Community Development in Hu Lambs During Early Life
Source: Front Microbiol. 2018 Mar 27;9:574. doi: 10.3389/fmicb.2018.00574 (PMC5881016; doi:10.3389/fmicb.2018.00574)
Supplement: Supplementary file 3 [file Table_3.docx]

**Table S3. The relative abundance (%) of phylum (with average relative abundance ≥ 0.5% in at least one age group) in Hu lambs with (S-ALF) or without (STA) alfalfa intervention** **at different ages.**

|  |  |  | Age (d) | | | | |  |  |
| --- | --- | --- | --- | --- | --- | --- | --- | --- | --- |
| Phylum | Group | B-10 | 17 | 24 | 38 | 45 | 66 | SEM | *P*-value^1^ |
| *Actinobacteria* | STA | 1.434 | 14.826 | 10.769 | 14.875 | 3.710 | 1.994 | 2.033 | .095 |
|  | S-ALF |  | 2.540 | 16.356 | 16.281 | 6.723 | 3.564 | 2.169 | .085 |
| *Bacteroidetes* | STA | 41.544 | 37.956 | 37.281 | 46.855 | 51.606 | 53.338 | 2.609 | .367 |
|  | S-ALF |  | 42.631 | 34.125 | 39.647 | 41.100 | 40.731 | 3.002 | .961 |
| *Chloroflexi* | STA | 0.568 | 0.022 | 0.118 | 0.013 | 0.132 | 0.198 | 0.107 | .019 |
|  | S-ALF |  | 0.193 | 0.335 | 0.042 | 0.166 | 0.916 | 0.138 | .039 |
| *Firmicutes* | STA | 35.467 | 42.227 | 47.917 | 35.689 | 37.894 | 36.875 | 2.711 | .556 |
|  | S-ALF |  | 46.974 | 41.489 | 39.160 | 45.655 | 49.962 | 3.097 | .512 |
| *Fusobacteria* | STA | 1.675 | 0.037 | 0.007 | 0.014 | 0.015 | 0.009 | 0.218 | .719 |
|  | S-ALF |  | 1.919 | 0.022 | 0.054 | 0.031 | 0.054 | 0.320 | .643 |
| *Planctomycetes* | STA | 0.005 | 0.008 | 0.350 | 0.001 | 0.005 | 0.020 | 0.034 | .084 |
|  | S-ALF |  | 0.014 | 0.011 | 0.001 | 0.033 | 0.962 | 0.143 | .222 |
| *Proteobacteria* | STA | 17.812 | 3.336 | 2.325 | 2.236 | 5.049 | 4.581 | 1.688 | .631 |
|  | S-ALF |  | 3.224 | 1.525 | 2.706 | 4.857 | 2.341 | 1.811 | .363 |
| *Spirochaetes* | STA | 0.060 | 0.207 | 0.150 | 0.139 | 1.044 | 2.356 | 0.216 | .001 |
|  | S-ALF |  | 0.973 | 5.115 | 1.846 | 0.867 | 1.032 | 0.601 | .398 |
| *Synergistetes* | STA | 0.042 | 0.143 | 0.103 | 0.074 | 0.311 | 0.145 | 0.050 | .394 |
|  | S-ALF |  | 0.965 | 0.563 | 0.049 | 0.088 | 0.094 | 0.123 | .342 |
| *Verrucomicrobia* | STA | 1.139 | 1.071 | 0.515 | 0.013 | 0.029 | 0.035 | 0.209 | .086 |
|  | S-ALF |  | 0.023 | 0.071 | 0.007 | 0.030 | 0.046 | 0.179 | .085 |

^1^*P*-value of age effect from d17 to 66.
