# Supplementary material for: Alfalfa Intervention Alters Rumen Microbial Community Development in Hu Lambs During Early Life
Source: Front Microbiol. 2018 Mar 27;9:574. doi: 10.3389/fmicb.2018.00574 (PMC5881016; doi:10.3389/fmicb.2018.00574)
Supplement: Supplementary file 4 [file Table_4.docx]

**Table S4. The relative abundance (%) of predominant genera (with average relative abundance ≥ 0.5% in at least one age group) in Hu lambs with (S-ALF) or without (STA) alfalfa intervention** **at different ages.**

|  |  |  |  | Age (d) | | | | |  |  |
| --- | --- | --- | --- | --- | --- | --- | --- | --- | --- | --- |
| Phylum | Genus | Group | B-10 | 17 | 24 | 38 | 45 | 66 | SEM | *P*-value^1^ |
| *Actinobacteria* | *Actinomyces* | STA | 0.271 | 0.130 | 0.088 | 0.039 | 0.025 | 0.019 | 0.014 | 0.508 |
|  |  | S-ALF |  | 0.519 | 0.030 | 0.066 | 0.028 | 0.035 | 0.063 | 0.272 |
|  | *Bifidobacterium* | STA | 0.228 | 3.286 | 0.343 | 1.822 | 0.452 | 0.313 | 0.482 | 0.185 |
|  |  | S-ALF |  | 1.166 | 3.108 | 3.454 | 1.253 | 0.422 | 0.496 | 0.228 |
|  | Uncalssified *Coriobacteriaceae* | STA | 0.669 | 11.308 | 10.107 | 12.900 | 3.132 | 1.552 | 2.052 | 0.090 |
|  |  | S-ALF |  | 0.746 | 13.064 | 12.636 | 5.225 | 2.946 | 2.283 | 0.060 |
| *Bacteroidetes* | Unclassified *Bacteroidales* | STA | 0.967 | 7.218 | 1.214 | 1.227 | 5.354 | 7.614 | 1.067 | 0.018 |
|  |  | S-ALF |  | 1.650 | 5.111 | 2.540 | 9.231 | 9.957 | 1.072 | 0.017 |
|  | Unclassified *Paraprevotellaceae* | STA | 0.054 | 0.189 | 0.180 | 0.170 | 0.328 | 1.159 | 0.099 | 0.004 |
|  |  | S-ALF |  | 0.218 | 0.411 | 0.231 | 0.451 | 0.375 | 0.053 | 0.415 |
|  | *CF231* | STA | 0.709 | 0.043 | 0.035 | 0.035 | 0.089 | 0.217 | 0.023 | 0.033 |
|  |  | S-ALF |  | 0.082 | 0.048 | 0.063 | 0.111 | 0.113 | 0.013 | 0.370 |
|  | *YRC22* | STA | 0.112 | 0.097 | 0.036 | 0.091 | 0.630 | 0.396 | 0.091 | 0.014 |
|  |  | S-ALF |  | 0.142 | 0.079 | 0.114 | 0.180 | 0.613 | 0.087 | 0.189 |
|  | *Bacteroides* | STA | 20.949 | 1.514 | 1.910 | 1.010 | 0.749 | 0.699 | 0.223 | 0.604 |
|  |  | S-ALF |  | 15.042 | 1.285 | 1.088 | 0.966 | 1.493 | 1.834 | 0.640 |
|  | Unclassified *BS11* | STA | 8.570 | 0.058 | 0.043 | 0.046 | 0.057 | 0.047 | 0.011 | 0.960 |
|  |  | S-ALF |  | 0.095 | 9.318 | 0.141 | 0.214 | 0.418 | 1.131 | 0.957 |
|  | Unclassified *p-2534-18B5* | STA | 0.021 | 0.261 | 0.057 | 0.021 | 0.192 | 0.022 | 0.045 | 0.758 |
|  |  | S-ALF |  | 0.806 | 0.005 | 0.065 | 0.030 | 0.035 | 0.131 | 0.291 |
|  | Unclassified *Porphyromonadaceae* | STA | 0.104 | 0.104 | 0.052 | 0.016 | 0.025 | 0.022 | 0.017 | 0.785 |
|  |  | S-ALF |  | 0.855 | 0.014 | 0.063 | 0.031 | 0.051 | 0.139 | 0.790 |
|  | *Porphyromonas* | STA | 2.278 | 0.261 | 0.280 | 0.132 | 0.098 | 0.124 | 0.045 | 0.616 |
|  |  | S-ALF |  | 2.844 | 0.155 | 0.289 | 0.084 | 0.368 | 0.316 | 0.378 |
|  | *Prevotella* | STA | 6.365 | 23.473 | 17.226 | 29.672 | 40.710 | 35.781 | 2.566 | 0.051 |
|  |  | S-ALF |  | 19.211 | 14.210 | 27.198 | 26.008 | 20.165 | 2.110 | 0.302 |
|  | Unclassified *S24-7* | STA | 0.871 | 4.362 | 15.982 | 14.319 | 3.294 | 7.183 | 1.682 | 0.024 |
|  |  | S-ALF |  | 1.398 | 3.293 | 7.738 | 3.687 | 6.615 | 0.919 | 0.210 |
| *Chloroflexi* | *SHD-231* | STA | 0.558 | 0.022 | 0.110 | 0.012 | 0.131 | 0.197 | 0.036 | 0.019 |
|  |  | S-ALF |  | 0.193 | 0.334 | 0.042 | 0.165 | 0.914 | 0.116 | 0.039 |
| *Firmicutes* | *Lactobacillus* | STA | 1.005 | 0.154 | 0.407 | 0.449 | 0.507 | 0.041 | 0.124 | 0.299 |
|  |  | S-ALF |  | 17.618 | 0.194 | 1.137 | 1.141 | 0.877 | 2.847 | 0.805 |
|  | *Streptococcus* | STA | 1.068 | 0.148 | 2.690 | 0.248 | 0.033 | 0.069 | 0.258 | 0.277 |
|  |  | S-ALF |  | 1.114 | 0.117 | 0.764 | 0.124 | 0.081 | 0.174 | 0.435 |
|  | Unclassified *Clostridiales* | STA | 4.013 | 5.717 | 4.483 | 4.677 | 8.540 | 8.329 | 0.843 | 0.187 |
|  |  | S-ALF |  | 3.745 | 8.264 | 4.372 | 10.279 | 16.740 | 1.321 | 0.005 |
|  | Unclassified *Mogibacteriaceae* | STA | 1.012 | 0.294 | 0.485 | 0.288 | 0.316 | 0.508 | 0.047 | 0.309 |
|  |  | S-ALF |  | 0.398 | 0.540 | 0.429 | 0.754 | 0.676 | 0.046 | 0.033 |
|  | *Mogibacterium* | STA | 0.292 | 0.194 | 0.288 | 0.167 | 0.391 | 0.617 | 0.060 | 0.047 |
|  |  | S-ALF |  | 0.337 | 0.525 | 0.292 | 0.691 | 0.651 | 0.061 | 0.075 |
|  | Unclassified *Lachnospiraceae* | STA | 4.923 | 3.019 | 6.905 | 3.555 | 5.432 | 7.222 | 0.606 | 0.013 |
|  |  | S-ALF |  | 3.622 | 4.152 | 6.910 | 8.291 | 7.395 | 0.748 | 0.108 |
|  | *Blautia* | STA | 0.309 | 0.276 | 0.320 | 0.192 | 0.262 | 0.268 | 0.022 | 0.601 |
|  |  | S-ALF |  | 0.515 | 0.317 | 0.255 | 0.231 | 0.411 | 0.037 | 0.085 |
|  | *Butyrivibrio* | STA | 1.270 | 3.084 | 2.262 | 5.833 | 10.422 | 5.917 | 1.046 | 0.036 |
|  |  | S-ALF |  | 2.979 | 7.806 | 11.302 | 7.277 | 8.696 | 1.117 | 0.095 |
|  | *Coprococcus* | STA | 1.562 | 0.017 | 0.019 | 0.013 | 0.030 | 0.042 | 0.003 | 0.021 |
|  |  | S-ALF |  | 0.021 | 0.022 | 0.024 | 0.045 | 0.044 | 0.005 | 0.257 |
|  | Unclassified *Ruminococcaceae* | STA | 1.992 | 1.944 | 1.140 | 0.675 | 1.739 | 2.649 | 0.214 | 0.004 |
|  |  | S-ALF |  | 3.672 | 2.262 | 1.757 | 2.289 | 3.119 | 0.380 | 0.439 |
|  | *Oscillospira* | STA | 1.421 | 0.312 | 0.176 | 0.139 | 0.210 | 0.266 | 0.028 | 0.076 |
|  |  | S-ALF |  | 0.511 | 0.162 | 0.260 | 0.285 | 0.299 | 0.041 | 0.145 |
|  | *Ruminococcus* | STA | 0.836 | 1.628 | 14.095 | 5.774 | 1.818 | 3.498 | 1.169 | 0.082 |
|  |  | S-ALF |  | 1.196 | 6.049 | 1.154 | 4.269 | 2.989 | 0.908 | 0.037 |
|  | Unclassified *Veillonellaceae* | STA | 0.505 | 1.860 | 5.438 | 4.812 | 1.006 | 1.673 | 0.999 | 0.722 |
|  |  | S-ALF |  | 0.798 | 0.563 | 0.873 | 1.598 | 1.537 | 0.201 | 0.069 |
|  | *Dialister* | STA | 5.326 | 3.396 | 1.102 | 4.663 | 0.835 | 0.520 | 0.581 | 0.007 |
|  |  | S-ALF |  | 2.656 | 0.916 | 3.481 | 1.315 | 0.758 | 0.495 | 0.802 |
|  | *Megasphaera* | STA | 1.508 | 2.367 | 1.036 | 0.646 | 0.900 | 0.297 | 0.294 | 0.142 |
|  |  | S-ALF |  | 0.464 | 0.520 | 0.431 | 0.382 | 0.433 | 0.055 | 0.981 |
|  | *Mitsuokella* | STA | 1.636 | 2.436 | 0.322 | 0.416 | 0.521 | 0.204 | 0.259 | 0.530 |
|  |  | S-ALF |  | 0.501 | 0.425 | 0.359 | 0.305 | 0.268 | 0.077 | 0.631 |
|  | *Selenomonas* | STA | 0.075 | 0.096 | 0.095 | 0.174 | 0.210 | 0.245 | 0.031 | 0.177 |
|  |  | S-ALF |  | 0.191 | 0.114 | 0.173 | 0.944 | 0.226 | 0.147 | 0.148 |
|  | *Succiniclasticum* | STA | 0.080 | 0.161 | 0.137 | 0.113 | 0.779 | 2.234 | 0.296 | 0.001 |
|  |  | S-ALF |  | 0.214 | 0.274 | 0.958 | 0.499 | 1.679 | 0.243 | 0.249 |
|  | *Bulleidia* | STA | 0.385 | 0.836 | 0.721 | 0.450 | 0.369 | 0.353 | 0.110 | 0.772 |
|  |  | S-ALF |  | 0.410 | 0.327 | 0.764 | 0.741 | 0.318 | 0.116 | 0.777 |
|  | *p-75-a5* | STA | 0.482 | 0.085 | 0.078 | 0.054 | 0.269 | 0.153 | 0.035 | 0.228 |
|  |  | S-ALF |  | 0.220 | 0.110 | 0.166 | 1.174 | 0.454 | 0.152 | 0.048 |
|  | *RFN20* | STA | 0.041 | 0.031 | 0.037 | 0.022 | 0.052 | 0.046 | 0.004 | 0.108 |
|  |  | S-ALF |  | 0.446 | 0.038 | 0.120 | 0.805 | 0.074 | 0.147 | 0.295 |
|  | *Sharpea* | STA | 3.070 | 12.884 | 4.836 | 1.343 | 1.462 | 0.246 | 1.584 | 0.029 |
|  |  | S-ALF |  | 3.993 | 6.502 | 1.761 | 0.665 | 0.273 | 1.117 | 0.048 |
| *Fusobacteria* | *Fusobacterium* | STA | 1.667 | 0.036 | 0.006 | 0.014 | 0.014 | 0.008 | 0.005 | 0.719 |
|  |  | S-ALF |  | 1.918 | 0.020 | 0.053 | 0.030 | 0.054 | 0.302 | 0.669 |
| *Planctomycetes* | Unclassified *Pirellulaceae* | STA | 0.003 | 0.008 | 0.350 | 0.001 | 0.005 | 0.020 | 0.042 | 0.084 |
|  |  | S-ALF |  | 0.014 | 0.011 | 0.001 | 0.033 | 0.962 | 0.179 | 0.222 |
| *Proteobacteria* | *Sutterella* | STA | 0.637 | 0.002 | 0.003 | 0.003 | 0.006 | 0.004 | 0.001 | 0.282 |
|  |  | S-ALF |  | 0.010 | 0.004 | 0.004 | 0.002 | 0.004 | 0.001 | 0.489 |
|  | *Eikenella* | STA | 3.776 | 0.009 | 0.005 | 0.003 | 0.004 | 0.000 | 0.001 | 0.261 |
|  |  | S-ALF |  | 0.002 | 0.001 | 0.000 | 0.002 | 0.003 | 0.001 | 0.495 |
|  | Unclassified *Rhodocyclaceae* | STA | 0.225 | 0.681 | 0.390 | 0.237 | 0.183 | 0.133 | 0.082 | 0.807 |
|  |  | S-ALF |  | 0.398 | 0.181 | 0.212 | 0.144 | 0.329 | 0.062 | 0.848 |
|  | *Campylobacter* | STA | 0.263 | 0.060 | 0.026 | 0.185 | 0.218 | 0.178 | 0.044 | 0.096 |
|  |  | S-ALF |  | 0.641 | 0.136 | 0.121 | 0.175 | 0.056 | 0.091 | 0.409 |
|  | Unclassified *Aeromonadaceae* | STA | 0.548 | 0.004 | 0.019 | 0.025 | 0.009 | 0.007 | 0.004 | 0.406 |
|  |  | S-ALF |  | 0.013 | 0.017 | 0.037 | 0.013 | 0.010 | 0.006 | 0.939 |
|  | Unclassified *Succinivibrionaceae* | STA | 0.039 | 0.125 | 0.077 | 0.084 | 2.477 | 2.843 | 0.654 | 0.078 |
|  |  | S-ALF |  | 0.056 | 0.099 | 0.091 | 2.926 | 0.119 | 0.712 | 0.234 |
|  | *Succinivibrio* | STA | 0.101 | 0.668 | 0.430 | 0.770 | 1.130 | 0.507 | 0.129 | 0.845 |
|  |  | S-ALF |  | 0.667 | 0.240 | 1.388 | 0.631 | 0.698 | 0.170 | 0.094 |
|  | Unclassified *Enterobacteriaceae* | STA | 1.325 | 0.164 | 0.194 | 0.113 | 0.159 | 0.064 | 0.036 | 0.835 |
|  |  | S-ALF |  | 0.156 | 0.082 | 0.117 | 0.087 | 0.249 | 0.042 | 0.351 |
|  | Unclassified *Pasteurellaceae* | STA | 2.200 | 0.048 | 0.021 | 0.011 | 0.014 | 0.008 | 0.005 | 0.643 |
|  |  | S-ALF |  | 0.044 | 0.022 | 0.015 | 0.010 | 0.014 | 0.004 | 0.079 |
|  | *Bibersteinia* | STA | 1.791 | 0.018 | 0.006 | 0.004 | 0.001 | 0.003 | 0.002 | 0.079 |
|  |  | S-ALF |  | 0.026 | 0.006 | 0.007 | 0.005 | 0.011 | 0.002 | 0.082 |
|  | *Moraxella* | STA | 0.924 | 0.018 | 0.011 | 0.001 | 0.003 | 0.002 | 0.002 | 0.452 |
|  |  | S-ALF |  | 0.014 | 0.004 | 0.006 | 0.003 | 0.014 | 0.003 | 0.631 |
| *Spirochaetes* | *Treponema* | STA | 0.053 | 0.184 | 0.095 | 0.121 | 0.951 | 2.324 | 0.258 | 0.001 |
|  |  | S-ALF |  | 0.949 | 5.089 | 1.829 | 0.822 | 1.001 | 0.735 | 0.400 |
| *Verrucomicrobia* | Unclassified *RFP12* | STA | 0.072 | 0.014 | 0.503 | 0.005 | 0.005 | 0.011 | 0.059 | 0.573 |
|  |  | S-ALF |  | 0.014 | 0.056 | 0.003 | 0.020 | 0.018 | 0.009 | 0.440 |
|  | *Akkermansia* | STA | 1.065 | 1.055 | 0.009 | 0.006 | 0.009 | 0.003 | 0.148 | 0.259 |
|  |  | S-ALF |  | 0.008 | 0.009 | 0.003 | 0.004 | 0.004 | 0.002 | 0.985 |

^1^*P*-value of age effect from d17 to 66.
