# Supplementary figures and images for: Alfalfa Intervention Alters Rumen Microbial Community Development in Hu Lambs During Early Life
Source: Front Microbiol. 2018 Mar 27;9:574. doi: 10.3389/fmicb.2018.00574 (PMC5881016; doi:10.3389/fmicb.2018.00574)

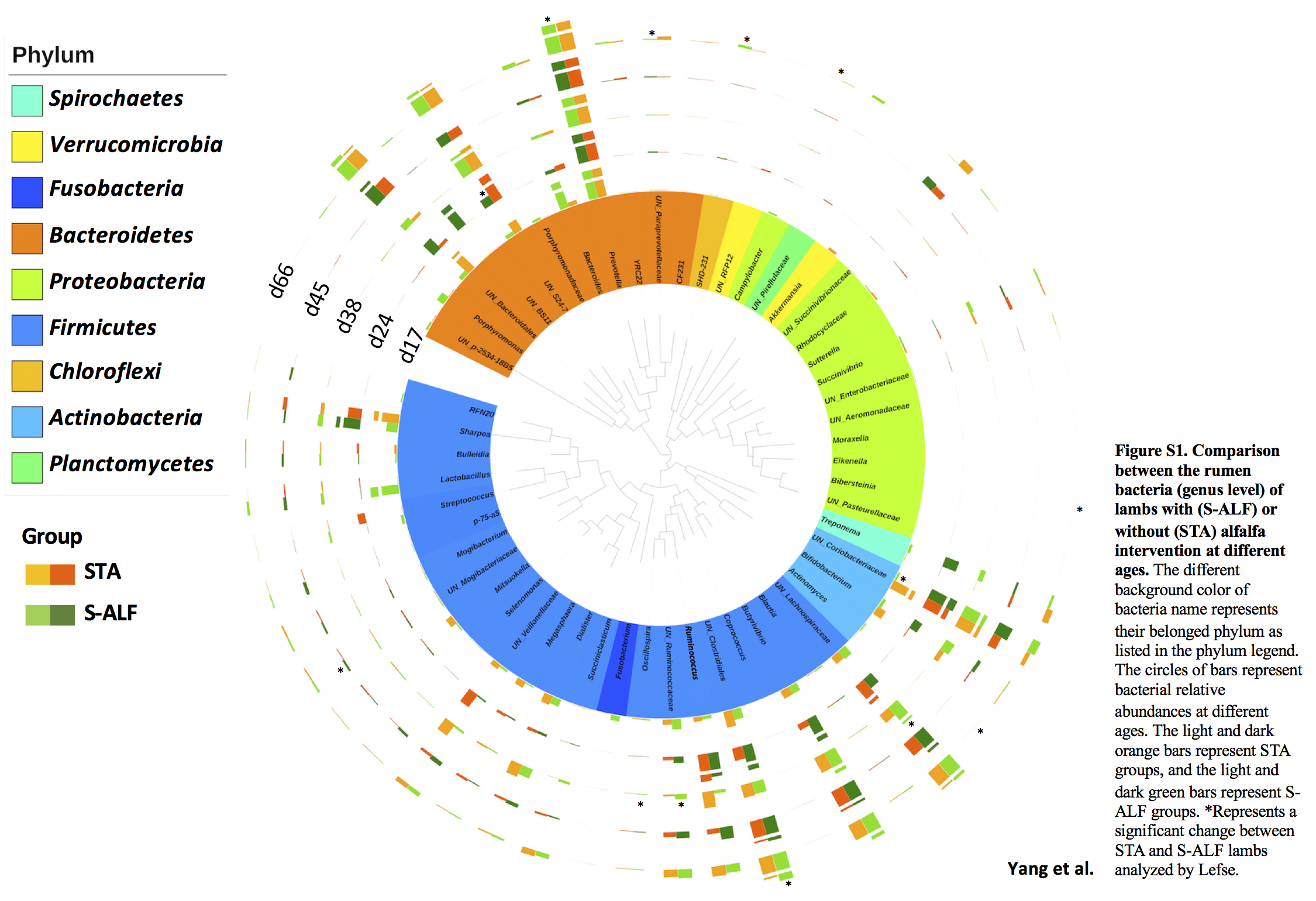

Supplement: Supplementary file 5 [file Image_1.JPEG]
